# Quantifying fluid retention following modern pain management in TKA: an observational study

**DOI:** 10.1186/s43019-024-00251-4

**Published:** 2024-12-16

**Authors:** Parkpoom Somrak, Aree Tanavalee, Srihatach Ngarmukos, Chotetawan Tanavalee, Chavarin Amarase, Nonn Jaruthien, Wirinaree Kampitak

**Affiliations:** 1 Advanced Arthritis and Arthroplasty Center, Department of Orthopaedics, Bumrungrad International Hospital, Bangkok, Thailand; 2 Biologics for Knee Osteoarthritis Research Unit, Faculty of Medicine, Chulalongkorn University, Bangkok, Thailand; 3 Department of Orthopaedics, Faculty of Medicine, Chulalongkorn University and King Chulalongkorn Memorial Hospital, Thai Red Cross Society, 1873 Rama IV Road, Bangkok, 10330 Thailand; 4 Department of Anaesthesiology, Faculty of Medicine, Chulalongkorn University and King Chulalongkorn Memorial Hospital, Thai Red Cross Society, Bangkok, Thailand

**Keywords:** Fluid balance, Total body water, TBW, Body weight, Fluid retention, Total knee arthroplasty, TKA

## Abstract

**Background:**

Body fluid retention after major surgeries, including total knee arthroplasty (TKA), is well documented in the literature. Currently, multimodal pain control protocols consisting of several medications together with early discharge protocol may magnify this adverse event after a patient’s discharge. However, no study has focused on the quantitative and chronological changes in body fluids following modern pain management protocols for TKA. The aim of this study was to investigate the perioperative total body water (TBW) change in patient undergoing TKA.

**Patients and methods:**

A consecutive series of 85 patients undergoing primary unilateral TKA, with uniform hospital admission, multimodal pain control, and rehabilitation protocol, had five consecutive multifrequency bioelectrical impedance analysis (BIA) scans; baseline, postoperative day 1 (POD 1), postoperative day 3 (POD 3), 2 weeks, and 6 weeks. Changes in TBW, body weight, corticosteroid-fluid retention dose–response relationship, and complications were evaluated.

**Results:**

Seventy patients completed all five scans and follow-ups. Female patients were dominant, with a mean age of 69.5 years. There were no perioperative complications. At 24 h, the mean total fluid input and output were 3695.14 mL and 1983.43 mL, respectively, with 1711.71 mL increments and a mean accumulative dosage of dexamethasone of 15.14 mg. The mean TBW increased by 2.61 L on POD 1 and continued to peak at 3.2 L on POD 3, then gradually decreased at 2 weeks and reached the baseline level at 6 weeks postoperatively. Similarly, the mean body weight increased to 2.8 kg on POD 1, reached the maximum point at 3.42 kg on POD 3, and returned to baseline at 6 weeks.

**Conclusions:**

Fluid retention following multimodal pain control in TKA increased from POD 1, peaked on POD 3, and gradually returned to the baseline at 6 weeks. With early discharge protocol, patient education regarding fluid retention after discharge should be considered.

## Introduction

Body fluid retention after major surgeries, including total knee arthroplasty (TKA), is well documented in the literature [7, 11]. Currently, perioperative fluid management in patients undergoing TKA varies among the treatment protocols of orthopedic surgeons and anesthesiologists. According to the change to an early discharge program after surgery, several treatment regimens, which may affect fluid balance, are applied, including, a shorter nothing-by-mouth (NPO) time, increased use of local anesthesia, less perioperative blood loss related to tranexamic acid usage, and minimizing surgical stress responses in an individual patient by using multiple drugs [17]. According to the concept of enhanced recovery after surgery (ERAS), the intravenous fluid is discontinued as soon as possible, with the expectation of a balanced fluid intake and output. However, with the use of multimodal pain control, some medications, especially corticosteroids and nonsteroid anti-inflammatory drugs (NSAIDs) widely used in most protocols in arthroplasty surgeries, can precipitate sodium and water retention [1, 4]. It is unclear whether the ERAS with modern pain management protocol may lead to over-fluid intake and postoperative fluid retention in TKA patients.

Body water retention in postoperative patients has been reported to increase respiratory complications and compromise cardiopulmonary functions [6, 24]. Interstitial fluid retention can induce local tissue inflammation and impair collagen reparation, thus causing surgical wound complications and delaying patients’ functional recovery [6, 9, 10, 24]. In the past decades, several studies aimed to establish the appropriate perioperative fluid management in patients undergoing joint arthroplasty, and the details and advantages of several protocols have been described [2, 12, 23]. However, none of the protocols included quantitative body fluid measurements to monitor patients’ total body water volume changes. Therefore, patients with lower limb swelling after TKA might relate to several causes, including fluid retention.

Bioelectrical impedance analysis (BIA) is a rapid, noninvasive tool developed to analyze body compositions based on the rate at which tiny electrical currents spread through different types of tissues in the body [20, 25]. In recent literature, the BIA has shown its accuracy and efficacy in clinical applications [3, 13, 20, 25], especially for evaluating body compositions in patients undergoing TKA [16, 19, 21]. However, only some studies have applied this tool to incorporate body compositions and the change of total body fluid as an outcome measure [19, 25]. Also, there has been an unknown incidence of perioperative fluid retention and its natural course of total fluid balance after TKA.

This study inspected the perioperative change of total body fluid in TKA patients under the ERAS with modern pain management protocol using a BIA as a quantitative measurement tool. The investigation duration for all changes was extended until 6 weeks postoperatively. The primary outcome was the total body water (TBW) change from preoperative to 6 weeks post-TKA. Secondary outcomes included changes in body weight, corticosteroid-fluid retention dose–response relationship, and other perioperative complications.

## Materials and methods

### Study design

The institutional review board (IRB) has approved this prospective observational study (COA no. 1483/2023 IRB no. 0574/66). From November 2023 to April 2024, a consecutive series of 85 adult patients scheduled for a uniform pattern of hospital admission and surgery with a single multimodal pain management and rehabilitation protocol were included in the study. All patients were admitted in the evening and had NPO after midnight, then underwent primary unilateral TKA on the morning of the following day as the first case of the surgical schedule. Selection criteria included age 45–75 years, end-stage knee osteoarthritis, and willingness to undergo several BIA scans preoperatively and postoperatively. Exclusion criteria were patients with cardiac pacemakers, volume-dependent conditions (such as severe aortic stenosis, cardiac arrhythmia, and pulmonary hypertension), end-stage renal disease, diabetes mellitus (DM) with HbA1C > 7%, chronic exogenous steroid use, and patients with body mass index (BMI) less than 19 or more than 38 kg/m^2^.

During admission, the patient underwent three consecutive multifrequency BIA scans (Inbody 770; Inbody USA; Cerritos, CA, USA, which utilized six frequencies for impedance measurement: 1, 5, 50, 250, 500, and 1000 kHz). By standing on the Inbody 770 scanner and holding the hand holders for 40–60 s, tetra-polar eight-point electrodes calculated the patient’s body fluid. All BIA scans were conducted in the morning on the day after the patient had the first urine void. The BIA scan was first tested before surgery and was considered the preoperative baseline data. During admission, the same patient underwent two BIA tests on postoperative day 1 (POD 1) and postoperative day 3 (POD 3). At the outpatient clinic, two additional scans were conducted in the morning of weeks 2 and 6 when the patients came for follow-up postoperatively.

### Surgery, anesthesia, and pain management

All surgeries were performed under opioid-sparing spinal anesthesia and a uniform perioperative management protocol. Preemptive analgesia included 650 mg paracetamol and 400 mg celecoxib (Celebrex^Ⓡ^) given orally 30 min before surgery. Regarding reduction perioperative blood loss protocol, 1000 mg tranexamic acid was given intravenously before tourniquet application, and another 500 mg was given intraarticularly after wound closure. All patients received 10–15 mg of intravenous dexamethasone, intraoperatively, with an additional 5 mg once a day on POD 1 and/or POD 2, according to individual anesthesiologist’s consideration and 4 mg of ondansetron for postoperative nausea and vomiting (PONV) prophylaxis. Two peripheral nerve blocks were applied in all knees, including a continuous adductor canal block (CACB) with 0.15% levobupivacaine continuously dripping at 5 mL/h for 48 h and a single-shot nerve block at the interspace between the popliteal artery and posterior capsule of the knee (iPACK), with 20 mL of 0.25% bupivacaine and 0.1 mg of epinephrine [14]. Before implantation, a periarticular infiltration (PAI) with a mixture containing 20 mL of 0.5% levobupivacaine, 30 mg of ketorolac, and 0.3 mg of epinephrine combined with saline solution for a total volume up to 80 mL was injected in all cases. No drain was applied in all knees, while the intraarticular injection of tranexamic acid was performed after complete wound closure.

Immediate postoperative medications included oral paracetamol (500 mg) every 6 h, 400 mg celecoxib (Celebrex^Ⓡ^), and 75 mg pregabalin (Lyrica^Ⓡ^) administered once daily for 2 days, postoperatively. Intraoperative fluid administration and output were determined by noninvasive monitoring parameters and managed by anesthesiologists. For pain rescue, 3 mg intravenous morphine every 3 h was administered if the postoperative pain scale was ≥ 5 for the first 48 h. Postoperatively, the patients were encouraged to have oral meals and fluid intake, the same as under their preoperative conditions. The postoperative intravenous fluid was restricted and the patient was discharged the morning of the next day. Discharge medications including 200 mg celecoxib (Celebrex^Ⓡ^) and 75 mg pregabalin (Lyrica^Ⓡ^) were given once daily for 14 days. A half tablet of Ultracet^Ⓡ^ (325 mg paracetamol and 37.5 mg tramadol) was prescribed as pro re nata (PRN) every 12 h if the pain scale after discharge was ≥ 5.

### Ambulation protocol and data collection

The early recovery protocol included the patient sitting with the foot dangling and knee extension exercises performed from the afternoon of the day of surgery (postoperative day 0, POD 0). From the morning of POD 1, rehabilitation with the patient standing and ambulating with a walking aid was instructed to increase activities. On POD 3, the patients were discharged based on goal-directed discharge criteria, including the ability to bear weight on the operated limb and walk for 50 m without foot and leg swelling. Patients’ demographic data, perioperative net fluid intake volume, and urine output were recorded and calculated. The cumulative steroid dosage each patient received was also put on record. The TBW from the preoperative period until 6 weeks post-TKA, changes in body weight, body fat percent, corticosteroid-fluid retention dose–response relationship, and other perioperative complications were evaluated and compared between preoperative and postoperative periods.

### Statistical analyses

Statistical analyses were completed using IBM® SPSS® Statistics version 29 (IBM Corp. 2022; Armonk, NY, USA). Descriptive statistics were used to analyze the distribution of categorical variables by mean ± standard deviation (SD). The generalized estimation equation model was used to evaluate the change in mean TBW, body weight, and body fat percentage measured at different periods. The corticosteroid dosage and fluid retention dose–response relationship were assessed using mixed-effects linear regression analysis.

## Results

**Fig. 1:**
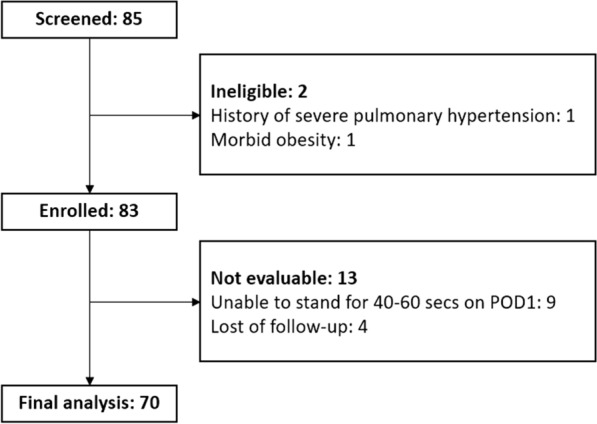
Flow diagram of participants screened and enrolled in the study

Among 85 screened patients, 83 were enrolled (2 were excluded because of a history of severe pulmonary hypertension and morbid obesity). During the study, nine patients could not stand on the BIA scanner for 40–60 s on POD 1, and four could not complete a followup and undergo a BIA scan at 6 weeks postoperatively. Therefore, 70 patients had completed all five scans and the final follow-up; the flow diagram is shown in Fig. 1. Female patients were dominant, with a mean age and BMI of 69.5 years and 29.94 kg/m^2^, respectively. The demographics and baseline parameters are presented in Table 1.

**Table 1:** Demographics baseline and perioperative parameters of participants undergoing total knee arthroplasty (TKA) and those who completed the study (*N* = 70)

	Participants (*n* = 70)	Min–max
Age (years) (mean ± SD)	69.51 ± 5.51	59–84
Sex		
- Female (number, percent)	58 (82.9%)	
- Male (number, percent)	12 (17.1%)	
ASA class		
- 1	22 (31.4%)	
- 2	44 (62.9%)	
- 3	4 (5.7%)	
BMI (kg/m^2^) (mean ± SD)	25.94 ± 3.48	21.4–35.02
Body fat (%) (mean ± SD)	38.1 ± 4.00	29–43.4
Baseline TBW (mean ± SD)	24.95 ± 3.29	20.28–31.53
Fluid input (mL) (mean ± SD)	3695.14 ± 293.35	3180–4260
Fluid output (mL) (mean ± SD)	1983.43 ± 327.19	1230–2500
Net fluid (mL) (mean ± SD)	1711.71 ± 461.83	810–2650
Dexamethasone dosage (mg) (mean ± SD)	15.14 ± 3.53	10.0–25.0
Implant brand and model		
- DePuy Synthes Attune	38 (54.3%)	Mean size femur 4, tibia 3
- Smith & Nephew Journey II	18 (25.7%)	Mean size femur 4, tibia 3
- Smith & Nephew Legion	14 (20.0%)	Mean size femur 4, tibia 2

*ASA* American Society of Anesthesiologists, *TBW* total body water, *BMI* body mass index

**Fig. 2:**
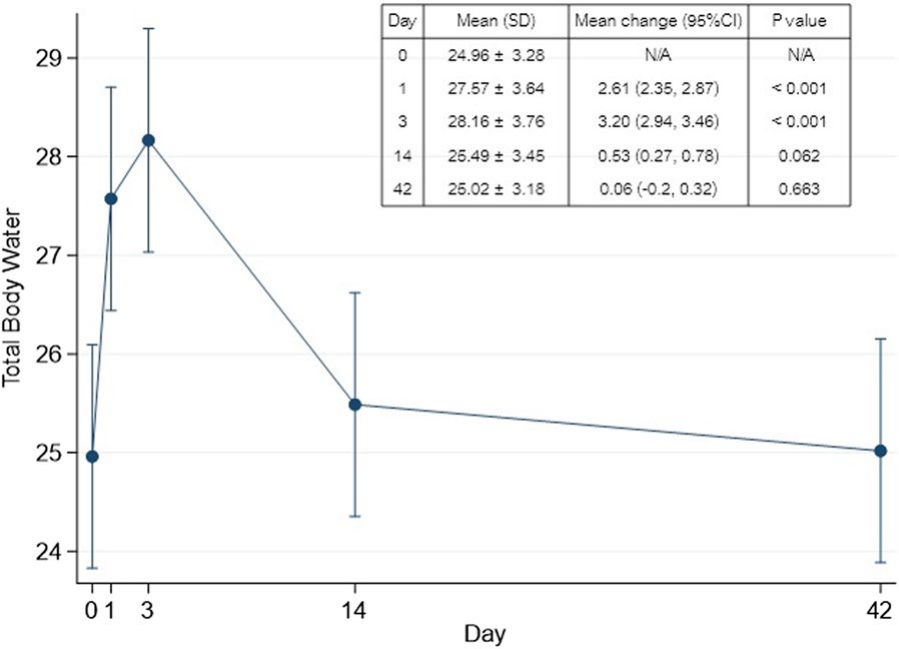
The mean ± standard deviation (SD) with a 95% confidence interval (CI) of total body water (TBW) showed significant changes from the baseline level (POD 0) on POD 1 and POD 3. It slowly decreased to the baseline level on POD 42

At 24 h after surgery, the mean ± SD of total fluid input and output of the studied group were 3695.14 ± 293.35 mL and 1983.43 ± 327.19 mL, respectively, with a total volume increase of 1711.71 ± 461.83 mL. On POD 1, the mean TBW increased by 2.61 L and continued up to 3.2 L on POD 3 with significant differences from the baseline. Although the TBW decreased at 2 weeks, it reached a level that was not different from the baseline value at 6 weeks postoperatively. Figure 2 shows the generalized estimating equation model results of the difference in TBW at various time points.

**Fig. 3:**
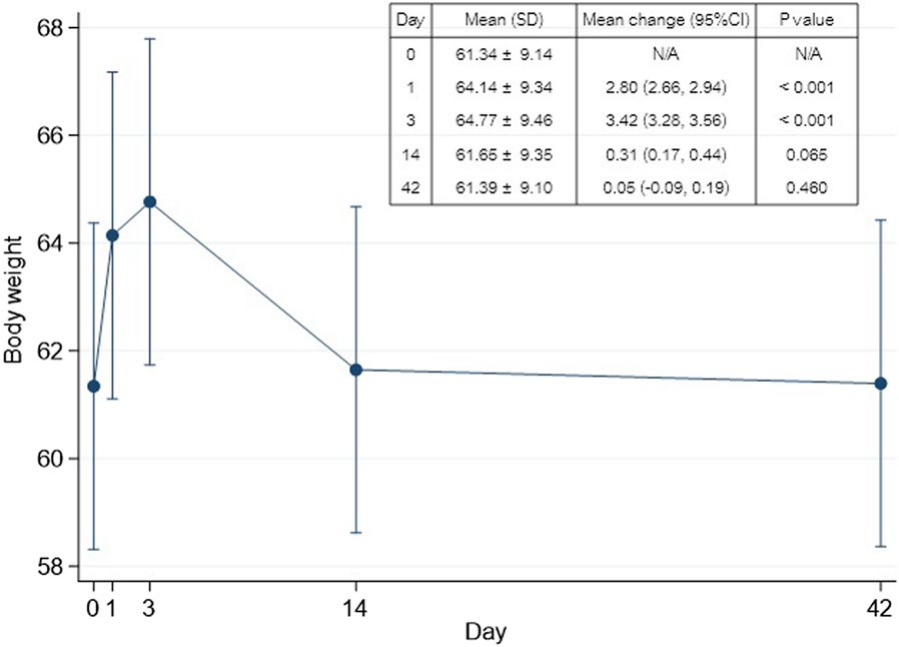
The mean ± standard deviation (SD) with a 95% confidence interval (CI) of body weight showed significant changes from the baseline level (POD 0) on POD 1 and POD 3. Similar to total body water (TBW) changes, it slowly decreased to the baseline level on POD 42

Regarding the change in body weight, the patients gained 2.8 kg on POD 1 and 3.42 kg on POD 3, with significant changes from baseline. The body weight remained slightly higher at 2 weeks and returned to baseline at 6 weeks postoperatively. Figure 3 shows the generalized estimating equation model results of the changes in body weight at different time points. In contrast, the body fat percentage decreased by 4.03%, *p* < 0.001) on POD 1, down to 4.89%, *p* < 0.001) on POD 3, and became similar to the baseline level (−0.17%, *p* = 0.567) at 6 weeks postoperatively, which conversely corresponded with TBW change.

The accumulative dosage of dexamethasone in each patient ranged between 10 and 25 mg, with a mean ± SD of 15.14 ± 3.53 mg. The patients’ perioperative parameters are summarized in Table 1. The mixed-effects linear regression analysis of accumulative dexamethasone dosage and water retention dose–response relationship showed that the beta coefficient was 0.1 with *p*-value = 0.008 for fluid volume change following TKA on POD 1. The correlations on other postoperative time points are presented in Table 2.

**Table 2:** The mixed-effects linear regression analysis of the accumulative dexamethasone dosage and water retention dose–response relationship showed a significant relationship (*p*-value, 0.008) for fluid volume change following TKA on POD 1

	Beta coefficient	95% CI	*p*-Value
Preoperative to POD 1	0.1	0.03, 0.18	*0.008*
Preoperative to POD 3	0.09	−0.01, 0.19	*0.071*
Preoperative to POD 14	0.05	−0.02, 0.12	*0.167*
Preoperative to POD 42	0.02	−0.01, 0.05	*0.111*

*POD* postoperative day

All patients underwent surgery without intraoperative and postoperative complications and were instructed to mobilize, ambulate, and walk as planned. No patient had nausea or vomiting during the perioperative period, and no patient required morphine for pain rescue.

## Discussion

Fluid retention after major surgeries has been demonstrated in the literature [1, 7, 11, 15], which is related to increased vascular permeability following surgical trauma to soft tissues and results in fluid collection in the third space [8, 22]. With an efficient pain management protocol and nausea/vomiting control in TKA, early recovery with early intravenous fluid discharge has become a routine postoperative scenario. It is the rationale that early mobility after surgery enhances the lower limb muscles in recovering to normal function. Therefore, early ambulation protocol in TKA combined with early discharge of intravenous fluid should enhance the returning fluid from the third space to the vascular system. As several medications used in multimodal pain management protocol are found to cause fluid retention, leg and foot swelling related to fluid retention was still common in TKA with early recovery protocol.

To our knowledge, this is the first observational study on quantifying fluid retention following ERAS in TKA with modern pain management, under a uniform pattern of hospital admission, surgery, and early recovery protocol. The present study found that, following a TKA with opioid-sparing analgesia, a multimodal protocol for pain control, and early discharge of intravenous fluid, there was a significant increase in TBW and body weight from POD 1 to the peak level on POD 3. These changes gradually decreased from POD 3 until there were no differences at 6 weeks postoperatively.

Usually, most patients report increased body weight after TKA, during early postoperative followup [5], which could be caused by several factors. While most surgeons and patients consider the weight of the TKA implant to be the critical factor, Gibon et al. reported [5] that the weight of all TKA prostheses and bone cement was more than that of removed bones and soft tissues, approximately 270 g. This value was less than 10% of the change in body weight from baseline to the immediate postoperative period. Therefore, the weight of the TKA implant is less likely to cause an increase in body weight after the surgery. Jennings et al. [12] studied body weight change from conventional intravenous fluid versus oral fluid administration for perioperative fluid management in TKA. The intravenous and oral fluid groups had increased body weight from preoperative baseline to 48 h postoperatively by 6.2 kg and 4.4 kg, respectively, which were higher than those found in our study. To explain the difference, our patients’ demographic data, especially BMI, was lower than Jennings’s study corresponding to the human body difference between the Asians and the Caucasians. Also, with restricted and early discharged intravenous fluid in our postoperative protocol, the total amount of perioperative cumulative intravenous fluid intake in our series was lower than that of Jennings’s study. Based on findings in both studies, fluid retention should be considered the primary factor in increasing body weight.

According to the study of Loyd et al. [18], the single-frequency BIA scan for lower extremity swelling following TKA provided reliability and precision, demonstrating significant limb swelling at 2 weeks and returning to baseline at 6 weeks postoperatively. Multifrequency BIA has been studied to investigate body composition parameters in various fields of medical practice [19, 25]. However, there have yet to be any specific studies regarding fluid retention in patients undergoing TKA. This study is the first to quantify TBW change and fluid retention incidence in the perioperative period following TKA. As the BIA demonstrated significantly increased TBW, but significantly decreased body fat percentage on POD1 and POD 3, we assumed that there was a reverse relationship between the total body water and the body fat percentage.

The present study demonstrated a dose–response relationship between the accumulative dosage of dexamethasone as part of multimodal analgesia and fluid retention after TKA. In our study, the average dosage of dexamethasone given during the perioperative period was 15.14 mg. The increasing dose of dexamethasone significantly correlated with increasing TBW from preoperative to POD 1 with a beta coefficient  of 0.1. However, the correlation was not found at the other time points. Regarding the prophylaxis of PONV, our anesthesiologists preferred different doses of dexamethasone combined with multimodal analgesia on POD 0, which became significant dose-related fluid retention on POD 1. Meanwhile, a low dose (5 mg per day) of dexamethasone given on POD 1 and/or POD 2 did not cause a significant effect on fluid retention compared with the preoperative period and POD 3 onward. Therefore, the dose and frequency of dexamethasone in multimodal analgesia in modern TKA should be reconsidered and in agreement between the surgeon and the anesthesiologist, especially in patients with volume-dependent conditions or with a higher risk of fluid retention.

All patients in the present study did not require morphine as pain rescue during the perioperative period using the early ambulation protocol was achieved. We found that it was related to a combined CACB, iPACK block, and PAI for pain control, which provided very efficient opioid-sparing pain control in TKA during admission. Thus, we could not evaluate fluid retention related to morphine usage in this study. Although a low dose of tramadol, a narcotic-derived painkiller contained in Ultracet^Ⓡ^, was prescribed at discharge, it was advised to be taken as a PRN medication with a limited dose (one tablet per day). As the fluid retention at follow-ups did not demonstrate any significant difference from baseline data, we might assume that a low dose of tramadol did not affect fluid retention.

The present study has some limitations. First, this study was a prospective observational study in which some factors affecting body fluid retention, especially the dose of steroids, were not controlled. Thus, further randomized controlled trials should be conducted to study the correlation between steroid dosage and water retention. Second, celecoxib, a cyclooxygenase-2 inhibitor NSAID, was administered to all patients at the same dose and duration as a main medication in multimodal pain control, and further randomized controlled trials for any correlation to fluid retention may be conducted. Third, we excluded the patients with baseline volume-dependent conditions. Therefore, the results of the present study may not be applied to this patient group. Lastly, the follow-up time points were based on the routine practice at our institution; thus, it might limit the evaluation of the exact period it took to resolve water retention. However, strengths of the present study include the prospective design to homogenize the studied group, including the NPO time, starting surgery time, anesthesia and pain control protocol, and ambulation protocol. With increased fluid retention in patients following contemporary multimodal anesthesia and pain control protocol for TKA, this information would remind orthopedic surgeons to be aware and pay more attention to fluid balance in TKA patients.

## Conclusions

In the era of early recovery after TKA and the use of combined medications in multimodal pain regimens, fluid retention and increased body weight occurred from the immediate postoperative period, approached the peak on day 3, declined at 2 weeks, and returned to baseline level at 6 weeks postoperatively. With the early discharge program after TKA, surgeons and patients should be aware of fluid retention and increased body weight, which could extend to 6 weeks postsurgery.

## Data Availability

The data used in our manuscript is available on request.
